# Carbon Dots-Mediated Fluorescent Scaffolds: Recent Trends in Image-Guided Tissue Engineering Applications

**DOI:** 10.3390/ijms22105378

**Published:** 2021-05-20

**Authors:** Mohan Vedhanayagam, Iruthayapandi Selestin Raja, Anara Molkenova, Timur Sh. Atabaev, Kalarical Janardhanan Sreeram, Dong-Wook Han

**Affiliations:** 1 CATERS Laboratory, CSIR-Central Leather Research Institute, Adyar, Chennai 600020, India; msvedhanayagam@gmail.com; 2 BIO-IT Fusion Technology Research Institute, Pusan National University, Busan 46241, Korea; rajaselestin@gmail.com (I.S.R.); molkenova@gmail.com (A.M.); 3 Department of Chemistry, Nazarbayev University, Nur-Sultan 010000, Kazakhstan; timur.atabaev@nu.edu.kz; 4 Department of Cogno-Mechatronics Engineering, College of Nanoscience & Nanotechnology, Pusan National University, Busan 46241, Korea

**Keywords:** carbon dots, fluorescent scaffold, mechanical strength, biodegradation, image-guided tissue engineering

## Abstract

Regeneration of damaged tissues or organs is one of the significant challenges in tissue engineering and regenerative medicine. Many researchers have fabricated various scaffolds to accelerate the tissue regeneration process. However, most of the scaffolds are limited in clinical trials due to scaffold inconsistency, non-biodegradability, and lack of non-invasive techniques to monitor tissue regeneration after implantation. Recently, carbon dots (CDs) mediated fluorescent scaffolds are widely explored for the application of image-guided tissue engineering due to their controlled architecture, light-emitting ability, higher chemical and photostability, excellent biocompatibility, and biodegradability. In this review, we provide an overview of the recent advancement of CDs in terms of their different synthesis methods, tunable physicochemical, mechanical, and optical properties, and their application in tissue engineering. Finally, this review concludes the further research directions that can be explored to apply CDs in tissue engineering.

## 1. Introduction

Regeneration of damaged or injured tissue is a significant challenge in tissue engineering applications [1,2,3,4]. An appropriate solution for the enhanced cellular phenomena would be designing tissue engineering constructs with suitable physicochemical, mechanical, and biological properties. Over the years, various tissue engineering constructs such as film, sponge, membrane, fiber, hydrogel, and 3D ink scaffold have been developed by many researchers to achieve success in tissue regeneration [5,6,7,8,9,10,11,12,13,14]. The biocompatible scaffolds closely mimic the three-dimensional (3D) architecture of native tissue, aiding cellular events such as attachment, proliferation, and differentiation [15,16]. Mostly, scaffolds are fabricated using natural and synthetic polymers through phase separation, self-assembly, gas foaming, and particulate leaching, emulsification, and electrospinning method, etc. [17,18,19]. The scaffolds of natural polymer provide higher biocompatibility and lower mechanical strength, whereas synthetic polymer-based scaffolds show enhanced mechanical strength with lower biocompatibility and biodegradability [11,14,16,19,20]. Hence, the researchers integrate both natural and synthetic polymers at the composition to mitigate the above problems [21,22,23]. However, most of the scaffolds are limited to clinical trials due to their lack of efficient, non-invasive techniques for in situ-tracking of the scaffold activities such as position, integration, and degradation after implantation. To overcome this problem, researchers employ various invasive (histology/scanning electron microscopy (SEM)) and non-invasive imaging methods (ultrasound imaging, magnetic resonance imaging, optical imaging, photoacoustic imaging, positron emission tomography (PET), and single-photon emission computed tomography (SPECT)) with or without fluorescent probes to monitor the scaffold’s activity during the in vivo tissue engineering application [24,25,26,27,28]. Among them, optical imaging is highly sensitive, safe (non-radioactive), low cost, easy to use, rapid, long-term, non-ionizing, continuous, and contact-free examination of in vitro and in vivo tissues [29,30,31]. However, an accurate result from the optical imaging technique is possible only in the tissue sample with planar surface morphology. In contrast, curving surface, degree of movement, light scattering, limited imaging depth, and inhomogeneity are the risk factors for obtaining good optical imaging. It has been documented that the scattering of near-infrared light is much lower than visible light [32]. Therefore, using a near infrared-based imaging probe in an optical microscope can facilitate deep imaging penetration with highly scattering tissues.

**Figure 1:**
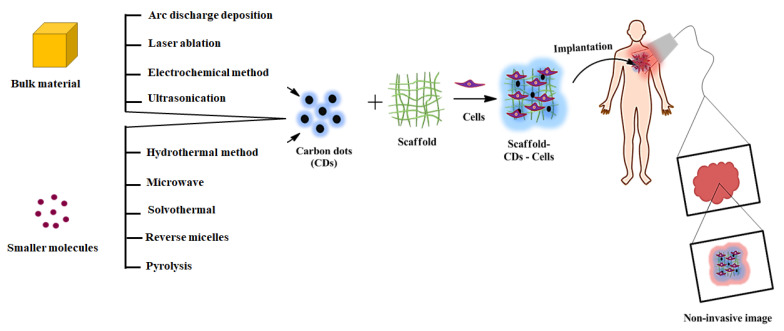
Schematic representation of different synthesis routes of CDs mediated scaffolds for the image-guided tissue engineering application.

Compared to conventional optical microscopy, advanced optical imaging methods such as two -or multi-photon confocal microscopy is considered the best one due to its high resolution, noise-free ability to focus on specific parts, and 3-D information of optically non-transparent living tissues at a deeper level (500–1500 µm) using NIR fluorescent probes [26,33,34]. The working principle of non-invasive imaging techniques is the physical interaction between the external light energy source and fluorescent probe-mediated implanted materials. Subsequently, the imaging system detects the energy change transmitted from the implanted materials generating a 3D image of reconstructed tissue (Figure 1). Several researchers have studied the biological events incorporating different fluorescent probes such as proteins, synthetic dyes, and iron oxide nanoparticle and semiconductor quantum dots (ex. selenium, tellurium, cadmium) to the scaffolds [20,35,36,37,38,39,40]. However, these fluorescence scaffolds suffer from rapid photobleaching and higher cytotoxicity due to heavy metal ions and incomplete biodegradation of semiconductor quantum dots. In the recent era, carbon dots (CDs) represent an attractive alternative to semiconductor quantum dots because of their unique carbon core structure, chemical inertness, low cost, excellent optical properties, better biocompatibility and biodegradability, higher water solubility and photo-stability, resistance to photo-bleaching, flexible surface modification, and environmental friendliness [41,42,43]. These exceptional features of CDs make them well suited for various applications, including chemical sensors, light-emitting diodes, photocatalysis, bioimaging, drug delivery, cancer theragnostic, and tissue engineering [44,45,46,47]. Carbon dots are a new class of carbon nanomaterials with 10 nm and are made up of sp^2^ and sp^3^ hybridized carbon atoms with zero-dimensional quasi-spherical structures. These carbon dots are classified into different types, including carbon quantum dots (CDs), graphene quantum dots (GQDs), carbon nanodots (CNDs), and carbonized polymer dots (CPDs), based on their variation in carbonization, graphitization, and polymerization in the synthetic process [48]. Incorporating various fluorescent carbon dots into scaffold endow higher surface area, tunable physicochemical, optical, and mechanical property, high stability and stimulating the biodegradation activities, and increasing biological interactions providing promising results in tissue engineering application [26,45,49]. In recent years, many reviews based on CDs have been reported in the research field, demonstrating different synthetic approaches, sensors, electronic and bioimaging applications [44,47,50,51,52,53,54,55,56,57,58,59,60,61,62]. However, very few reports are available to elucidate the versatility of CDs in non-invasive tissue engineering applications. Highlighting the recent progress in developing fluorescent CDs mediated tissue engineering platforms, giving an insight into CDs’ potential role in the field could be informative. Hence, in this review, we have provided a deep insight into the recent development of CDs mediated fluorescent scaffold in tissue engineering applications describing different synthetic approaches of scaffolds, fluorescence mechanisms, and the associated physicochemical properties.

## 2. Synthesis of Carbon Dots

Researchers are developing various synthetic approaches, either top-down or bottom-up methods, to prepare high-performance CDs [44,47]. The top-down approach involves the breaking down of bulky materials into nanoparticles sized less than 10 nm. The synthetic approaches, including arc discharge deposition, laser ablation, electrochemical carbonization, and ultrasonication are classified into the top-down synthetic method of CQDs. In the bottom-up approach, CDs are synthesized by building upon small molecules, including hydrothermal, pyrolysis, solvothermal, microwave, reverse micelles, etc.

### 2.1. Top-Down Approach

#### 2.1.1. Arc Discharge Deposition Method

**Figure 2:**
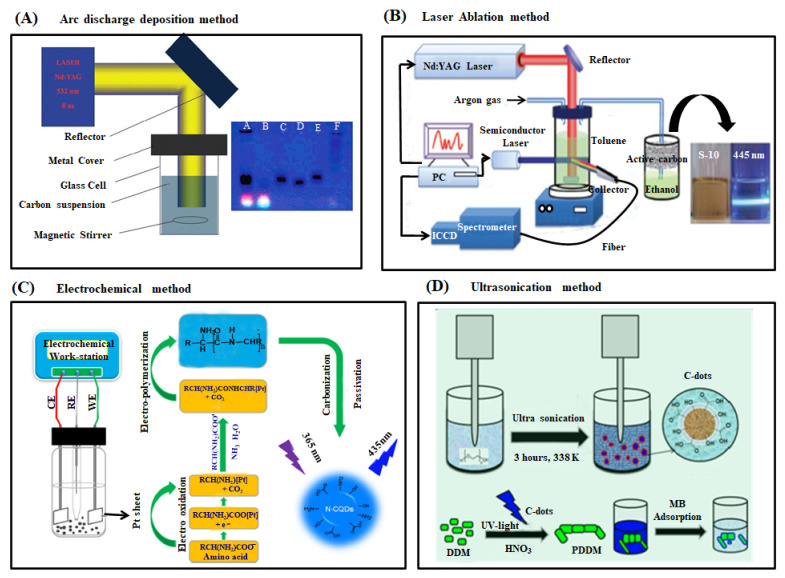
(**A**) Schematic representation of arc discharge deposition experimental setup (left) and photograph of fluorescent CDs after purification via gel electrophoresis method [64,67] (**B**) Schematic diagram of laser ablation synthesis of CDs and the visual appearance of CDs solution before and after UV light irradiation (Nd-YAG: Neodymium-doped yttrium aluminum garnet) [68] (**C**) Schematic depiction of the experimental setup and the possible reaction mechanism of N-CDs preparation from amino acids under alkaline condition through electrochemical method (two Pt sheets and Ag/AgCl were used as working (WE), counter (CE) and reference electrodes (RE), respectively). The synthesized N-CDs exhibited a strong blue fluorescence under UV light illumination at 365 nm [69]. (**D**) Schematic illustration of producing CDs through ultrasonication method, which was used as a catalyst for synthesis of PDDM from DDM monomer for the application of methylene blue removal from the aqueous solution [70].

Arc discharge deposition is a specialized method to prepare nanomaterials in an inert atmosphere [63]. In this method, two graphitic rods are used as cathode and anode electrodes and are separated by few millimeter distances. The bulk carbon sources are vaporized by higher energy arc plasma in a high electric current. The resulting vapors reorganize around the anode surface and form CDs, which are then deposited on the cathode electrode surface. In 2004, Xu and his research group unexpectedly obtained fluorescent carbon dots as a side product when they attempted to purify single-walled carbon nanotubes from the crude soot by arc discharge method [64]. They observed three types of materials, such as longer nanotube, irregular with short tubular, and fluorescent material, when the obtained suspension was employed for traditional gel electrophoresis (Figure 2A). The fluorescent material separated was found to have a size of 1 nm with 1.6% of quantum yield. The surface of the prepared CDs was surrounded by carboxylic groups, which led to a higher water solubility with different fluorescent emissions such as blue-green, yellow, and orange at 365 nm excitation. Similarly, Bottini et al. obtained fluorescent carbon dots as a minor product during the preparation of single-walled carbon nanotubes by arc discharge method [65]. The prepared carbon dots exhibited a smaller size with significant oxygen content emitting a higher level of fluorescence (blue to yellow-green) without surface passivation. Notably, the fluorescence property of CDs is highly dependent on the molecular weight of the fraction. The quantum yield of carbon dots could be improved by altering the bandgap of fluorescent carbon dots via heteroatom doping in the carbon core. For instance, Dey and his coworkers prepared N, B atom doped graphene quantum dots (GQDs) from graphite, boron powder, diboron vapor, and NH_3_ as C, B, and N sources, respectively, through arc discharge followed by the chemical scissoring method [66]. They observed that N, B doped GQDs exhibited a higher fluorescent emission with independent excitation due to their smaller sizes and higher crystallinity. The CDs obtained by this method registered a lower yield with several impurities, which were found difficult to be removed.

#### 2.1.2. Laser Ablation Method

The laser ablation method utilizes laser light to irradiate the bulk carbon source to separate fluorescent carbon dots from the carbon target’s surface [71]. Laser ablation is a rapid, effective, and simple approach for the preparation of CDs. Sun et al. prepared CQDs, for the first time, with non-fluorescence characteristics through laser ablation of a graphite powder in the presence of water vapor and argon gas as a carrier at the conditions of 900 °C and 75 kPa [72]. They passivated the sample’s surface with simple organic species via acid treatment to observe a stronger fluorescence emission. The diamine-terminated oligomeric poly-(ethylene glycol) (PEG 1500 N) passivated CQDs exhibited a smaller size (5 nm) with a 4–10% quantum yield. Similarly, Hu et al. prepared CQDs by laser irradiation of graphite powder dispersed in organic solvents (Figure 2B). They reported that the surface states of CQDs could be altered to attain tunable light emission by choosing the appropriate organic solvents. The luminescence property of CQDs was attributed to the surface states associated with many carboxylate groups on the surface of carbon dots [73]. Li et al. synthesized CQDs from the nano-carbon suspension in simple solvent through laser ablation method [67]. Notably, the laser ablation method produces less yield of CDs with utilization efficiency, and hence their various potential applications are still limited [71].

#### 2.1.3. Electrochemical Synthesis

Electrochemical graphitization is a powerful approach to prepare CDs from the bulk carbon material precursor because the method does not require a surface passivation and purification process. The other advantageous properties are easy manipulation, low cost, and high output. For example, Deng et al. prepared CDs from low-molecular-weight alcohols through electrochemical carbonization under some primary conditions [74]. Two platinum sheets and calomel electrodes were used as a working, auxiliary, and reference electrode, respectively, in that process. The graphitization degree and particle size of the prepared CDs were highly dependent on the applied potential. Interestingly, the size of CDs gradually increased with the expanding applied potential. During the formation of CDs, a large number of alcohols was oxidized, causing cross-linking and dehydration through the construction of active intermediates such as free radicals and carbonium ions. The prepared CDs expressed size-dependent fluorescence emission with 15.9% of quantum yield and significant toxicity to human cancer cells. Niu et al. rapidly synthesized multifunctional nitrogen-doped carbon quantum dots (N-CQDs) from amino acids via a simple three-electrode system (Figure 2C). N-CQDs, displaying a highly stable photoluminescent and electroluminescence property with 46.2% quantum yield, have been extensively used in cellular imaging, fiber staining, and Fe^3+^ ion detection applications [69]. Bao et al. prepared monodispersed CDs from the carbon fiber surface through the electrochemical etching method [75]. It was found that the size of CDs gradually decreased when the applied potential was increased. The smooth surface of CDs was converted into a rough surface at a higher applied potential, resulting in numerous burs on the surface of carbon fiber and more production of CDs from the solution.

#### 2.1.4. Ultrasonication

Ultrasonication is a simple, rapid, easy to manipulate, and low-cost method for the preparation of nanomaterials. In this method, the electrical signal converts into mechanical energy via sound waves that can rupture the bulk carbon materials. The constant distraction of sound waves facilitates the dissolution of smaller solid carbon precursors into a liquid phase and forms the emissive carbon dots [76]. For instance, Part and his coworker produced carbon dots from food waste material through the ultrasonication method at room temperature [77]. The prepared carbon dots presented a higher water solubility due to the presence of a large number of oxygen-containing functional groups on the carbon dots’ surface. Also, these CDs demonstrated lower cytotoxicity leading to their practical use in bioimaging applications. Similarly, Li et al. prepared highly water-soluble CDs from the active carbon source through the simple hydrogen peroxide-assisted ultrasonication method [78]. These CDs exhibited more hydrophilicity, photostability, broad fluorescence emission (visible to the near-infrared range), and outstanding up-conversion fluorescent properties. Recently, Maruthapandi et al. prepared CDs from the PEG-400 precursor via ultrasonication treatment [70]. Polymer coated CDs were used as free radical initiators for the polymerization of the 4,4′-diaminodiphenylmethane monomers (Figure 2D).

### 2.2. Bottom-Up Approaches

#### 2.2.1. Hydrothermal Method

**Figure 3:**
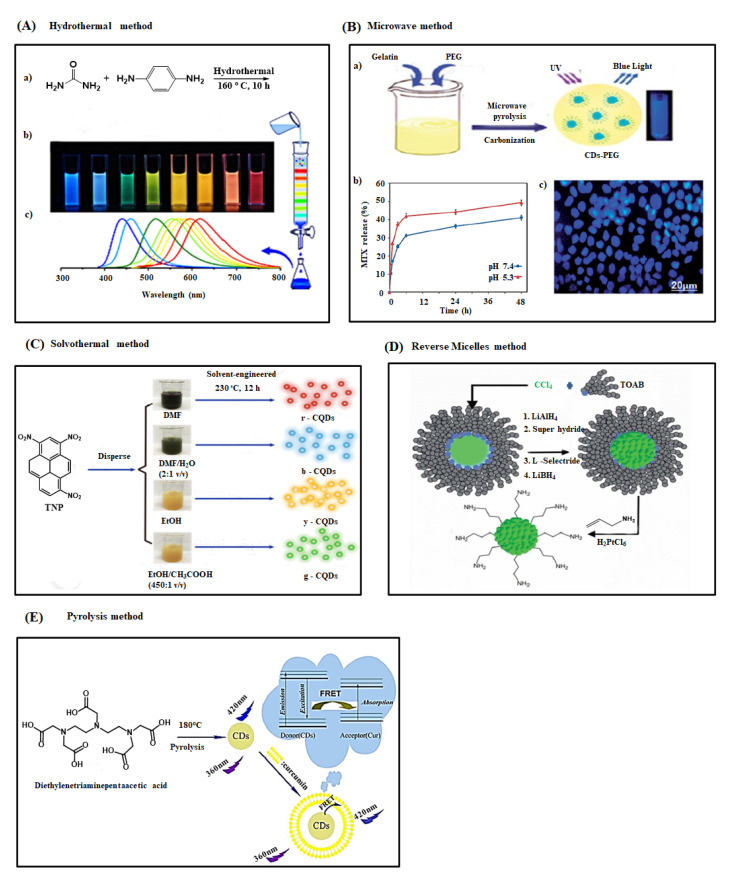
(**A**) (a) Hydrothermal synthesis of CDs with different fluorescence color (mixture) from urea and p-phenylenediamine (1:1) and it’s separated through silica column chromatography via changing the solvent polarity (b) Photo image of separated different CDs samples under single wavelength of UV light (365 nm) (c) Corresponding fluorescence emission spectra of CDs samples in the range of blue to red region due to decrease the band gap of CDs by increasing the degree of surface oxidation [80]. (**B**) (a) Schematic representation of the synthesis procedure of PEG coated CD via microwave carbonization method, (b) pH dependent MTX release behavior from MTX-PEG-CDs and, (c) fluorescence imaging of MCF-7 cells with MTX-PEG-CDs carrier under 405 nm excitation after 3 h [84] (**C**) Preparation of multicolor CDs from TNP as a carbon source through solvothermal method. DMF or EtOH are the major solvents while H_2_O or CH_3_COOH are the secondary solvents [85]. (**D**) Graphical depiction of the preparation of fluorescent CDs with different size from CCl_4_ and TOAB by changing the strength of reducing agent used in reverse micelle method [86]. (**E**) Synthesis of CDs through pyrolysis method using diethylenetriaminepentaacetic acid as a carbon source and their plausible mechanism for fluorescence emission and response towards curcumin (Cur) [87].

The hydrothermal method is widely used to prepare homogeneous and high-quality CDs due to their low energy consumption, inexpensive instrumentation, and easy control over the reaction parameter [79]. A recent study by Ding et al. used urea and p-phenylenediamine as the precursors for synthesizing multicolor CDs through a one-step hydrothermal method (Figure 3A). They separated the CDs through silica column chromatography and found its broad emission in the range of blue to red colors on a single UV light wavelength with 35% of quantum yield [80]. Likewise, Sahu and his coworkers prepared CDs from a cheap and readily available natural source, orange juice, using the hydrothermal method [81]. The synthesized CDs had a particle size of around 2.5 nm with spherical morphology and higher homogeneity. The CDs showed excellent water solubility because of the hydroxyl, epoxy, carbonyl, and carboxylic acid groups on the surface of carbon dots. Besides, CDs exhibited a 26% quantum yield with less toxicity against L929 and MG-63 cell lines, proving its candidature in cellular imaging applications. In the same way, Zhan et al. prepared nitrogen-doped carbon quantum dots (N-CQDs) with 85.3% yield from chitosan as a precursor via the hydrothermal method [82]. The synthesized N-CQDs exhibited a smaller size (2–10 nm) with a 6.6% quantum yield. These fluorescent N-CQDs were used to detect the Fe^3+^ ions in a lower concentration of 1.57 µM. Recently, Zhu et al. synthesized 2–6 nm-sized CDs with 80% of quantum yield from the citric acid and ethylenediamine precursors via the hydrothermal method [83]. These highly fluorescent CDs were applied as fluorescent ink to detect Fe^3+^ ions in the biosystems with a detection limit of 1 ppm.

#### 2.2.2. Microwave Method

Carbonizing small precursors using the microwave method is an efficient route for the synthesis of CDs compared to conventional methods. The technique has various advantages: uniform heating, rapid reaction rate, higher yield with immense purity, and cost-effectiveness [88,89]. Amorphous CDs with different sizes and luminescent properties were prepared by Zhu et al. by simply changing the microwave reaction time [90]. Liu et al. synthesized CDs from glycerol and 4,7,10-trioxo-1,13-tridecylenediamine precursor via a microwave method.

These fluorescent CDs demonstrated a 2–7 nm particle size with a 12% of quantum yield [91]. Another report by Filho et al. reported fluorescent carbon dots with 23.6% quantum yield synthesized from lemon biomass and onion juices via a microwave method [92]. The synthesized CDs had a spherical morphology with higher monodispersity and a particle size of about 6.15 nm. These fluorescent CDs were used to detect riboflavin specifically in multivitamin/mineral supplements. The quantum yield of CDs was further enhanced through surface coating or passivation on their surface. Interestingly, Arsalani et al. employed gelatin and polyethylene glycol as a carbon precursor and surface passivating agent, respectively, to prepare fluorescent PEGylated carbon dots (CDs-PEG) through the microwave pyrolysis route [84]. The CDs-PEG exhibited a smaller size (6 nm) with higher water solubility, photostability, and quantum yield (34%) compared to the CDs prepared from the gelatin source alone (Figure 3B). Subsequently, the fluorescent CDs-PEG was loaded with methotrexate (MTX) drug molecules to prepare CDs-PEG-MTX-based nanocarriers as an efficient drug delivery system. The prepared CDs-PEG-MTX nanocarriers proved higher biocompatibility towards MCF-7 cells and showed a site-selective and prolonged drug release capability at cancer tissue environment conditions. Xu and his coworker prepared aspirin-coated CDs using aspirin and hydrazine as the carbon precursors through one-step microwave treatment [93]. The 2–5.5 nm-sized aspirin-coated fluorescent CDs exhibited 23% of quantum yield, higher biocompatibility, and anti-inflammation activity. Furthermore, these fluorescent CDs internalized into the RAW 264.7 cells and HeLa cells in large quantity and showed less cytotoxicity suggesting a potential candidate for the cellular and bioimaging application.

#### 2.2.3. Solvothermal Method

In the solvothermal method, a solvent is used to prepare CDs through the solvothermal carbonization of smaller organic molecules [48,94]. For instance, Li et al. demonstrated a one-step solvothermal method using spinach as the precursor for synthesizing NIR-light emissive carbon dots (CDs). The synthesized CDs showed a smaller particle size (3–11 nm) with better water solubility, maximum fluorescence emission at 680 nm with 15–34% of quantum yield, higher photo-stability, and resistance to metal ions in a physiological environment, lower cytotoxicity, and excellent labeling capability. These multifunctional CDs make their potential use in NIR light-based bioimaging applications [95]. Zhan et al. prepared multicolored CDs from 2,4,6-trinitropyrene in different organic solvents (DMF, H_2_O, EtOH, and CH_3_COOH) through the solvothermal method [85]. They validated differently colored fluorescence CDs (blue, green, yellow, and red) with higher quantum yield (6.4–59%) by simply changing the solvent ratio (Figure 3C).

#### 2.2.4. Reverse Micelles Method

Synthesis of CDs through reverse micelle is a promising method due to their ability to control their size, shape, and surface chemistry [96]. Linehan et al. prepared allylamine terminated CQDs from carbon tetrachloride (CCl_4_) and tetraoctylammonium bromide (TOAB) through microemulsion synthesis route and reported that the prepared CQDs had a crystalline structure with spherical morphology (size 2–6 nm) and a higher photostability with 27% of quantum yield (Figure 3D). They found that the size of allylamine terminated CQDs could be tuned by changing the concentration of the reducing agent in the preparation step [86]. The research group of Prikhozhdenko et al. employed a hexan-Brij L4 and hexan-Brij L4-aqua solution-based two and three-component systems to prepare hydrophobic fluorescent CNDs by solvothermal assisted reverse micelles method [97]. They discovered that adding a small number of water molecules in the process increased the CNDs’ quantum yield by 0.6%, altering the structure of the Brij L4 molecule. Kwon et al. prepared graphitic carbon quantum dots through one-step carbonization of glucose molecules in water-in-oil reverse micelles [98]. An aqueous solution of glucose was added into the AOT surfactants-containing decane solution to form water-in-oil reverse micelles by the procedure. After heating these micelles at 160 °C, the transparent micelles turned to orange color due to the formation of oligosaccharides via condensation-polymerization of the glucose molecules. Subsequently, the continuous evaporation of water molecules induced the micelles to reach a critical supersaturation point and resulted in the simultaneous carbonization of the oligosaccharides and the in situ surface passivation. Tuning the size of fluorescent CQDs was achieved by changing the water: AOT surfactant molar ratio.

#### 2.2.5. Pyrolysis Method

The pyrolysis process has been widely used to prepare CDs via carbonization of organic molecule-based precursors at a higher temperature in an inert atmosphere. During the carbonization process, precursors involve in several reactions, including dehydration, polymerization, carbonization, and carbon dot formation. The method has several advantages: simple, rapid, inexpensiveness, and reduction of greenhouse gas emissions and water pollution [99,100]. For example, Shi et al. used diethylenetriaminepentaacetic acid (DTPA) as the precursor for the preparation of fluorescent CDs following one-step pyrolysis treatment (Figure 3E). They placed DTPA in a ceramic crucible and heated it to 180 °C in a thermal mantle for 5 min. They observed the change in sample color from white to dark brown-yellow while cooling the reaction temperature to 25 °C, which indicated the formation of fluorescent CDs. They discovered that the prepared CDs detected cur in the range of 0.74–5.18 µg mL^−1^ in the drug sample [87]. Likewise, Lai et al. prepared polyethylene glycol-coated carbon dots from glycerol and PEG through a one-step pyrolysis method and informed that they displayed higher stability and biocompatibility with 20% quantum yield [99]. Also, Yin et al. used citric acid monohydrate and ethylenediamine to prepare nitrogen-doped carbon dots through the pyrolysis method [101]. The synthesized 5 nm-sized N-CDs exhibited spherical morphology with a higher photostability and 88% of quantum yield. They further analyzed the influence of reaction time, temperature, and the molar ratio of precursor in the stability and fluorescence property of N-CDs. We have compiled a list of synthetic approaches of carbon dots from various precursors with their properties and applications in Table 1.

Overall, synthetic approaches such as electrochemical, hydrothermal, solvothermal, and microwave methods are potential in tuning the size, shape, and fluorescence properties of produced CDs. Because these methods highly facilitate the particle growth individually using the prospective regulation and uniform reaction condition.

## 3. Properties of Carbon Dots

The unique physicochemical characteristics and mechanical, electronic, magnetic, and optical properties of CDs are due to their smaller size, larger surface area, quantum confinement, and surface edge effect [44,52]. In this portion, we highlight the significant properties of CDs and demonstrate how they are related to tissue engineering applications [45].

### 3.1. Mechanical Strength Concept

Designing scaffolds with suitable mechanical properties is an essential criterion in tissue engineering applications as the cell adhesion, proliferation, and differentiation are highly dependent on the mechanical property of the scaffold [25,102]. Scientific studies have reported that the scaffold materials, including fiber, film, and hydrogel, exhibited substantially increased mechanical properties due to the presence of CDs [103,104,105,106,107]. For example, Ghorghi et al. revealed that the incorporation of CQDs into the polycaprolactone- captopril (PCL-CP) scaffold led to an enhanced tensile strength (22.09 ± 0.06 MPa) and Young’s modulus (2.83 ± 0.23 MPa) than that of pure PCL scaffold (tensile strength 6.86 MPa and Young’s modulus 0.15 MPa). The result was attributed to the decreasing fiber diameter of the PCL-CP scaffold in the presence of CQDs [108]. Another study by Omidie and his coworker described that the tensile strength, percentage elongation, and Young’s modulus of CD-chitosan scaffold significantly increased to 79 ± 4.4 MPa, 9.1 ± 0.22, and 2.88 ± 0.17 GPa, respectively, by the addition of 1% CDs to pure chitosan scaffold (64 ± 3.1 MPa, 8.6 ± 0.19, and 2.26 ± 0.20 GPa, respectively) [109]. Abolghasemzade et al. reported that BC-CQDs-Si NPs-SF scaffold exhibited around 2-fold higher elongations than BC scaffold due to the uniform distribution of CQDs-Si NPs-SF composite, which triggered a stronger coherence between BC and CQDs-Si NPs-SF composite [110]. Wang et al. exposed that CQDs enhanced the elastic modulus of ALg and CNF hydrogel by 4.7 and 1.5-fold, respectively [111]. The higher mechanical property was attributed to the formation of a hydrogen bond between the CQDs and hydrogel. Similarly, Li et al. observed an increase in the mechanical properties of the hydrogel by the presence of CQDs [112]. Yan et al. found that p-phenylenediamine functionalized CQDs substantially improved Young’s modulus of SF-PLA scaffold (1610 kPa), which was higher than that of SF-PLA scaffold (466 kPa) [49]. Notably, the size of the nanoparticles can alter the mechanical properties of the tissue engineering scaffold [11]. A research group of Safaie et al. provided a comprehensive study to determine the influence of CQDs with different sizes on the mechanical strength of resulting CDs blended polypropylene (PP) fiber. They documented that 1% of 2 nm-sized CQDs exhibited higher mechanical strength than the pristine fiber because of the stronger interaction between the CQDs and PP [113]. Shamy et al. prepared various concentrated CQDs that incorporated PVA nanocomposite films by the casting technique. They found an increase in tensile strength and Young’s modulus and decreased tensile strain with the gradually increasing concentration of CQDs in PVA film [114]. Incorporating CQDs in PVA film increased the packing density of the resulting nanocomposite film leading to the reduction of lattice strain under external tensile stress. Furthermore, the CQDs facilitated the charge transfer complex (CTC) by forming intra or intermolecular hydrogen forces that could prevent the PVA fibers from sliding over each other [115]. Similarly, Gogoi and his coworkers observed a concentration-dependent mechanical reinforcement in CDs-HAp-PU based nanocomposite film. The uniform distribution of CDs on the HAp surface contained many oxygen-related functional groups, which crosslinked polyurethane covalently or noncovalently to improve the mechanical strength [116]. In brief, the detailed literature survey suggested that CDs have a solid potential to enhance the mechanical strength of the various types of scaffolds for tissue engineering applications.

### 3.2. Optical Properties of CDs

**Figure 4:**
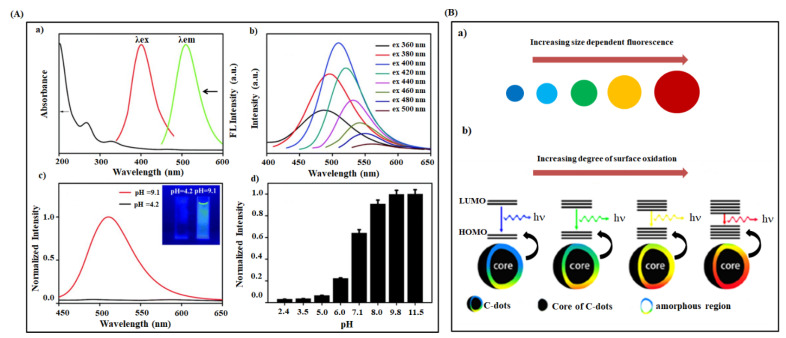
(**A**) Optical properties of CDs (a) UV-Visible absorption, fluorescence excitation and emission spectra of CDs prepared from phenylboronic acid (b) Fluorescence emission spectra of CDs at different excitation wavelength varied from 360 to 500 nm with 20 nm increments (c) Fluorescence spectra of CDs at acidic and alkaline condition (Inset: Photos of CDs solution at pH = 4.2 and pH = 9.1 under 365 nm UV light) (d) Normalized fluorescence intensity of CDs at various pH values [118]. (**B**) (a) Plausible model for the tunable fluorescence emission of CDs with increasing size and (b) degrees of surface oxidation [80].

Owing to the broad excitation and emission wavelength, the optical properties of CDs have received significant attraction and excitement in several biomedical applications, including cellular imaging, cancer therapy, drug delivery, and deep-tissue imaging [44,52,117]. Most of the synthesized CDs show two absorption peaks in the UV-visible region, viz. 230–280 nm and 400–650 nm. The absorption peak of the lower wavelength corresponds to the π-π* transition of the carbogenic core. In contrast, the peak at higher wavelength attributes to the n-π* transition of C=O and C=N bonds of the surface functional groups [118,119]. These absorption regions may shift to a longer region upon the surface modification of CDs with passivation agents or doping by N, S-atom in carbogenic core [118,120,121]. The excited CDs exhibit a broad fluorescence emission in the range of ultraviolet to near-infrared regions due to their distinctive chemical structure [95]. Several researchers have reported that the fluorescence property of CDs is highly dependent on the size of CDs [77,122,123]. For example, Yu et al. prepared differently sized CDs by changing the input laser in the laser ablation method, which showed a size-dependent fluorescence emission in the visible region due to the quantum confinement effect [68]. Also, Peng et al. formulated different fluorescence emissive carbon dots (blue, green, and yellow), changing their sizes (1–4 nm, 4–8 nm, and 7.11 nm), which was resulted in the variation in reaction temperature [124]. Recently, Kim and his group observed size-dependent fluorescence emission in CDs associated with the shape or edge state variations [125]. Li et al. synthesized CQDs successfully via an alkali-assisted electrochemical method. They exposed that CQDs with different sizes such as 1.2 nm, 1.5–3 nm, and 3.8 nm produced an emission spectrum in UV (350 nm), visible (400–700 nm), and near-infrared (800 nm) regions, respectively [126]. Also, CQDs emitted the light in the range of 325 to 425 nm, when they were excited by the longer wavelength light from 500 to 1000 nm. These observations indicated that the CQDs had a size-dependent fluorescence emission property along with the unique up-conversion phenomenon due to their quantum-sized graphite structures. Generally, the up-conversion event is a kind of emission process. The material involves sequential absorption of multiphoton, leading to light emission at a lower wavelength than the excitation wavelength [127]. Deng et al. prepared different sized carbon dots by changing the applied potential in the electrochemical carbonization method [74]. The resultant CDs showed excitation and size-dependent fluorescence emission by their variable sizes and different emissive sites on the CD surface. Also, some researchers reported that the surface functional groups of CDs have a strong influence on the optical properties of CDs [128,129]. Zheng and his coworkers produced an enhanced blue fluorescence emitting CDs (reduced form) from their weak green fluorescent CDs (oxidized form) through sodium borohydride reduction [130]. In this study, they observed a strong blue shift in the fluorescence of CDs (from 520 nm to 440 nm), a higher quantum yield of 24%, and a longer photostability of 6 months. The enhanced blue fluorescence was attributed to the change in CDs’ surface functional groups, i.e., carbonyl and epoxy groups to hydroxyl groups through the reduction reaction. Hu et al. prepared in situ surface-modified fluorescent CDs by dispersing graphite powder in organic solvents in laser irradiation method [73]. They suggested that suitable surface states of CDs leading to their higher fluorescence could be generated by choosing the proper solvents. The fluorescence property of PEG200N coated CDs was ascribed to the presence of carboxylate ligands on the CDs’ surface. In another similar research work, an enhanced quantum yield effect (32%) was observed for the PEG-coated CDs-mSiO_2_ nanocomposite [99]. Remarkably, CDs exhibit a strong fluorescence in pH 6–7, and the fluorescence intensity decreases either at higher acidic or basic conditions [131,132,133]. Wang et al. reported that the CQDs blended PVA material imparted tunable fluorescence property to the polymer [118]. The prepared CQDs-PVA composite showed higher pH sensitivity displaying an excitation-dependent emission spectrum (Figure 4A). The pH-responsive fluorescence of CDs was attributed to the changes in surface carboxyl groups such as protonation and deprotonation in acid and basic environment, respectively. According to the previous studies, the shift in the fluorescent peak for different excitation wavelengths was observed due to the synergic effects such as different size and emissive sites on CDs [75,134,135,136]. A few related emissive CDs undergo excitement and fluorescence at a particular excitation wavelength ensuring their excitation-dependent emission spectra [75,135]. These fluorescence spectra of CDs can mirror the distribution of different emissive sites on CDs’ surfaces [136]. Li et al. prepared NIR light-emissive CDs from spinach precursors through a solvothermal method exhibiting two different fluorescence emission peaks in the ranges of 400–600 nm (blue to yellow) and 600–750 nm (red) [95]. The first peak corresponded to carbogenic core state emission (excitation-dependent characteristic). The later one was assigned to the surface molecular state emission (excitation-independent characteristic) of spinach-coated CDs. They reported a higher level of surface defects on CDs’ surface is possible through more surface oxidation reactions [75]. Notably, various surface defects can make different emissive sites on CDs leading to variation in the respective emission spectra [137,138,139]. For instance, Ding et al. prepared CDs with multicolor emission from blue to red by hydrothermal method. They ascribed red fluorescence shift to the difference in the degree of surface oxidation of CDs (Figure 4B) [80]. The higher degree of surface oxidation on CDs leads to a decrease in the bandgap and, as a result, causes a redshift in fluorescence. Also, Bao et al. informed that a size-dependent red shift in emission spectra was observed for the CDs synthesized from the carbon fiber through the electrochemical method due to the available oxygen-related surface functional groups [75].

Based on these reports, several researchers have demonstrated the fluorescence property of CDs through various possible explanations such as size dependence (quantum confinement effects), surface defects, surface states, degree of oxidation, surface functional groups, surface passivation, fluorophores with various degree of π conjugation, and recombination of electron-hole pairs localized within small sp^2^ carbon clusters surrounded within a sp^3^ matrix [140,141,142,143,144]. However, the origin of the CDs’ fluorescence property is still not clearly understood due to the variation in carbon sources, synthetic methods, and complex chemical structure, which complicated to develop a unified theory. Meanwhile, there is no doubt that the excellent fluorescent property of CDs has great potential in tissue engineering applications.

## 4. CDs Mediated Scaffold for Tissue Engineering Application

The design and fabrication of suitable tissue constructs with three-dimensional structures are attractive in tissue engineering applications. The fundamental elements required for the fabrication of tissue constructs are appropriate cells and the scaffolds combined with signaling and therapeutic molecules. The scaffold plays a vital role in converting cells into tissue and acts as a template for regulating cellular function (cell adhesion, growth, proliferation, differentiation) and extracellular matrix deposition [3]. In addition, the scaffold allows the transfer and removal of the nutrients of toxic metabolites/by-products from in and out of the tissues. The scaffold’s physicochemical properties, mechanical strength, and biocompatibility depend on the three-dimensional architecture and chemical composition [11,12,15]. Hence, the fabricated scaffold should possess the following properties for tissue engineering application (1) high porosity with interconnected pore structure, (2) larger surface area, (3) suitable mechanical strength, (4) better biocompatibility, and (5) controlled biodegradation for sufficient cell-scaffold interaction [18,19,20]. Meanwhile, carbon dots are integrated into various natural and synthetic polymer-based scaffolds to enhance tissue engineering applications [145]. A list of carbon dots-based tissue engineering scaffolds or constructs has been demonstrated in Table 2, along with their fabrication methods and physicochemical properties.

Ren et al. prepared p-phenylenediamine functionalized CQDs mediated silk fibroin-PLA (CQDs-SF-PLA) nanofibrous scaffolds with better mechanical properties for the application of cardiac tissue engineering and nursing care [49]. The incorporation of CQDs into SF-PLA scaffolds significantly enhanced the cell adhesion, proliferation, and mRNA expression of cardiac genes (Tnnc1, Tnnt2, Cx43, and Atp2a2) without any external electrical supply. Ghorghi et al. fabricated captopril/CQDs/polycaprolactone (CP-CQDs-PCL) nanocomposite scaffold through the electrospinning method and observed that CQDs in the scaffold led to the decrease in the fiber diameter due to its electrically conductive nature [108]. The mechanical strength of the prepared scaffold increased while decreasing the fiber diameter. The scaffold with 0.5% CQDs significantly increased MG-63 cell adhesion, proliferation, and ALP activity due to their reduced fiber diameter and increased surface hydrophilicity, which was found to be adequate for bone tissue engineering application. Li et al. designed a CQDs (2 mg/mL) incorporated carboxymethyl chitosan blended oxidized dextran (CMCS-CQDs-ODex) hydrogel composite to show multifunctional properties such as self-healing, stretchable, compressive property, and biofilm formation inhibition [112]. This chemically cross-linked hydrogel exhibited higher antibacterial activity as the CQDs can produce intracellular ROS in bacterial cells resulting in their lysis. Besides, the hydrogel augmented higher granulation tissue formation and collagen deposition during skin regeneration.

Abolghasemzade et al. fabricated surface-modified CQDs (BC or PVA)-Si NPs-SF based nanofiber through spray printing and electrospinning methods for the applications of skin tissue and hair regeneration [110]. PVA-CQDs-SiNPs-SF nanofiber exhibited a higher elongation property, better antibacterial activity, and enhanced the NIH-3T3 cell proliferation and migration, leading to accelerated in vivo skin regeneration (93.3% re-epithelization on day 14), which was significantly larger than that of BC-CQDs-Si NPs-SF nanofiber (~65%).

**Figure 5:**
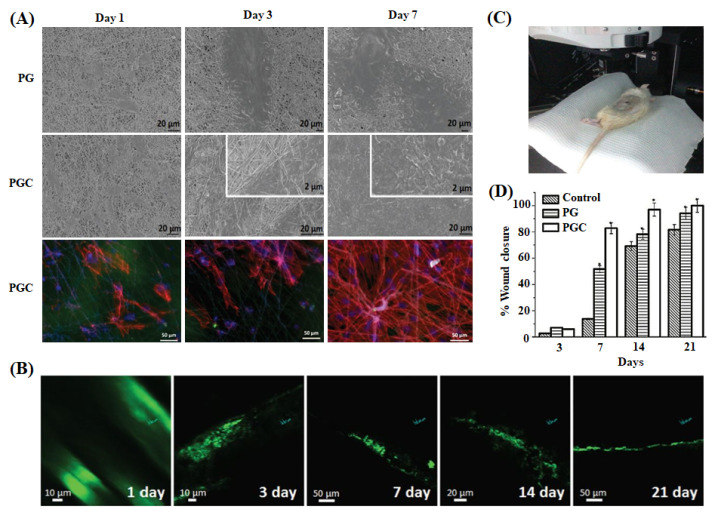
(**A**) SEM micrographs of primary fibroblast cells grown on PCL-gelatin nanofibers (PG) and PCL-gelatin nanofibers-CQDs (PGC) on 1, 3, and 7 days. Rhodamine cytoplasmic staining (red) and DAPI nuclear staining (blue) images of cells over PGC scaffolds are also shown. (**B**) Two-photon microscopy images of PGC nanofibers implanted on a full-thickness wound created in an adult Wistar rat model and (**C**) the set-up of two-photon microscopic imaging of an anesthetized live rat. (**D**) Percentage of wound area reduction in the control and scaffold (PG and PGC) treated rat groups after 3, 7, 14, and 21 days. * indicates *p* < 0.05 compared with control (*n* = 3) [146].

Pal et al. developed multifunctional CNDs containing fluorescent nanofibrous scaffold (polycaprolactone-gelatin matrix-CNDs) for performing long-time non-invasive imaging of cell-scaffold interaction during the in vivo skin tissue regeneration [146]. In this study, CNDs imparted fluorescent property to the scaffold, preserved the fiber morphology, and stimulated fibroblast cell adhesion, migration, and proliferation. The fluorescent scaffold was implanted in the wound area to monitor the regeneration process regularly via two-photon microscope images, as shown in Figure 5. Initially, the scaffold was completely adsorbed on the wounded area’s surface. After some time, the scaffold showed a non-uniform fluorescent pattern due to the scaffold’s infiltration of surrounding tissue cells. The continuously decreasing scaffold coverage and thickness indicated penetration of scaffold into the wound area, supporting the re-epithelialization on the scaffold surface. Further, the morphology of the scaffold became considerably discontinuous throughout the regeneration process, suggesting rapid biodegradation. The existence of highly stable fluorescent CND in the scaffold helped for the long-time non-invasive cell-scaffold interaction to analyze the skin regeneration kinetics.

**Figure 6:**
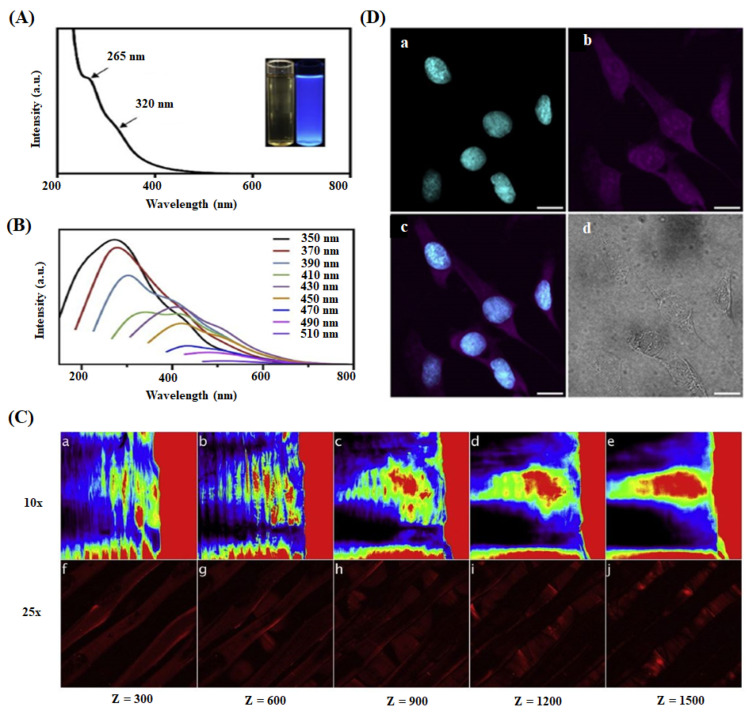
(**A**) UV-Visible absorption spectrum of CQDs in aqueous dispersion. (**B**) Photoluminescence spectra of CQDs with variable excitation wavelengths. (**C**) Multi-photon confocal microscope imaging of 3D printed collagen-CQDs scaffold at variable depths (z = 0–1500 µm) using different objectives 10× (a–e) and 25× (f–j). (**D**) Two-photon microscopic imaging of CQDs treated cells using a 780 nm pulsed laser: (a) Cyan represents nuclei stained with DAPI and (b) magenta indicates cytoplasm and nuclei interacting with CQDs. (c) Overlay of (a) and (b) and (d) brightfield image of the cells. scale bar is 20 µm [26].

Kandra et al. reported that CQDs loaded chitosan film, prepared for skin regeneration application, showed a better antioxidant property than pure chitosan leads [147]. Shafiei et al. developed poly (ε-caprolactone)-polyvinyl alcohol-tricalcium phosphate-carbon dots (PCL-PVA-TCP3-CDs) nanofibrous scaffolds through the electrospinning method for the effective bone tissue engineering application [145]. In this study, 1.0 wt% of CDs and TCP3 into the scaffold displayed a higher hydrophilic nature, better cell proliferation, and ALP activity leading to the enhanced osteogenic differentiation due to the combined effect of CDs and TCP3. Gogoi et al. fabricated CDs-peptide blended tannic acid-polyurethane (CDP-*f*-PU) based non-invasive scaffolds through the chemical cross-linking method and found that the scaffolds exhibited higher biocompatibility, osteoconductivity, and bone differentiation ability in bone tissue regeneration [148]. In an alternative study, they developed mechanically stable and biologically active CDs/hydroxyapatite/polyurethane scaffolds for the same biomedical application [116]. The prepared scaffold showed remarkable calcium/phosphorus (Ca/P) ratio value of 1.69, which was close to natural bone material (Ca/P = 1.67). The CDs-mediated scaffold exhibited higher load-bearing ability and enhanced alkaline phosphate activity against MG-63 cells than the scaffold alone. Dehghani et al. prepared CQDs-PLA fluorescent scaffold with high biocompatibility and photostability for the real-time monitoring and visualization of cell-scaffold interaction and scaffold degradation process during tissue regeneration in living organisms [26]. They visualized the scaffold’s 3D structure in various penetration depths (z = 0 to 1500 µm) under the biomimetic conditions. The images of space and separated PLA fiber layers of the scaffold at different z values are shown in Figure 6. The results suggested that CQDs could absorb the longer wavelength light (780 nm, 35 mW) at higher penetration depth and subsequently emit a strong fluorescence signal to the detector. They identified that the skin regeneration was highly dependent on the pH of injured or damaged tissue [150,151,152].

**Figure 7:**
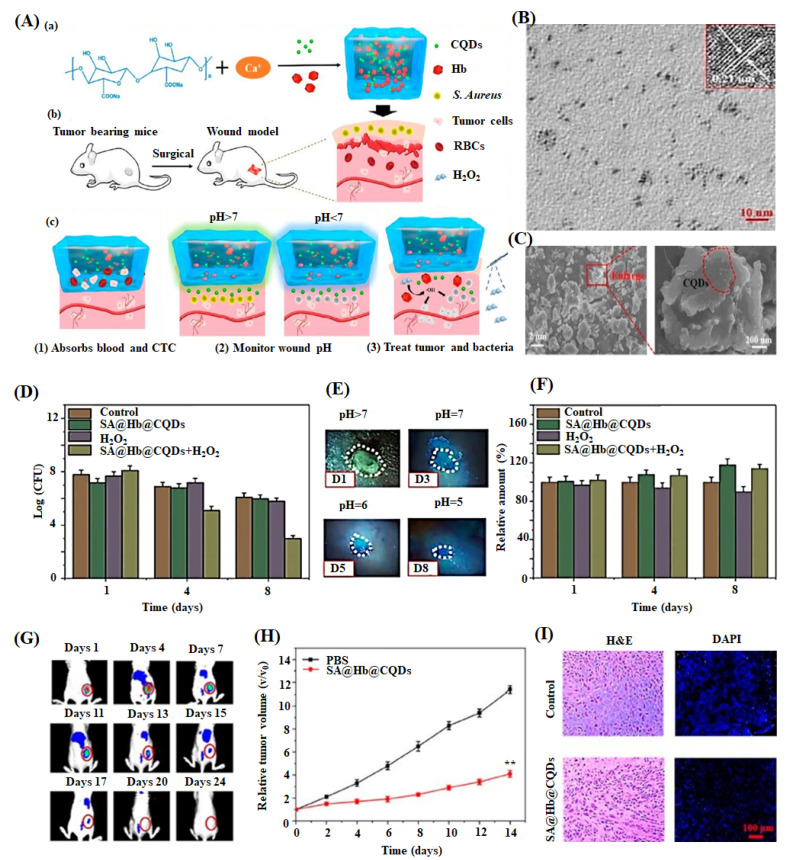
(**A**) Schematic diagram of preparation of sodium alginate hydrogel containing hemoglobin and CQDs (SA@Hb@CQDs). (a) Establishment of post-operative tumor model, (b) corresponding status of wound by the treatment of material, and (c) CTC-circulating tumor cells are shown. (**B**) TEM image of CQDs (inset: HRTEM images of CQDs) with scale bar 10 nm. (**C**) SEM images of SA@Hb@CQDs with its partial enlarged view. (**D**) Bacterial quantity of the *S. aureus*-infected wounds after treatment with samples at days 1,4, and 8. (**E**) Photographs of wounds treated with SA@Hb@CQDs followed by H_2_O_2_ under UV light at day 1, 3, 5, and 8. (**F**) Quantification of fibrinogen positive curves of the samples at the injured region on 1, 4, and 8 of post-days treatment. (**G**) Dynamic fluorescence imaging of indocyanine green-SA@Hb@CQDs treated mice on different days 1–24. (**H**) Tumor volume change curves and (**I**) H&E and DAPI images of tumors after treatment with control (PBS) and SA@Hb@CQDs are displayed [149].

Omidi et al. developed 1.0 wt% of CDs incorporated chitosan-based hydrogel through solvent casting method. The hydrogel, exhibiting a high pH sensitivity and enhanced optical and mechanical properties, monitored the damaged tissue and augmented the skin regeneration (16 days) through better antibacterial activity and biocompatibility [109]. Similarly, Zhang et al. studied sodium alginate-hemoglobin-CQDs (SA-Hb-CQDs) based pH-sensitive hydrogel for tissue engineering and cancer therapy applications [149]. In this work, they applied the hydrogel to the bacterially infected wound area and subsequently added H_2_O_2_ reagent to activate the antibacterial activity. The wound healing process was monitored under UV light conditions (Figure 7). Initially, the wound displayed green color under UV light, suggesting that the wound pH was above 7 due to higher bacterial infection. When H_2_O_2_ was injected into the wound site, the color changed to blue as the pH of the wound area decreases via the reduction of bacterial quantity through the Fenton reaction. These observations indicated that CQDs identified the wound site’s pH and accelerated skin regeneration through better antibacterial activity.

## 5. Future Perspectives and Challenges

A comprehensive review of fluorescent carbon dots mediated tissue engineering constructs has indicated that the multiphoton microscope extensively improved the non-invasive imaging of in-vivo tissue engineering scaffold. Furthermore, the CDs have contributed to enhancing the biofunctionality and biodegradability of the scaffolds. The longer emissive wavelength, higher photo-stability, better physicochemical and mechanical properties, broad pH sensitivity, deep tissue penetration, enhanced antibacterial activity, and excellent biocompatibility are the major factors for the success of CDs in tissue engineering applications. Meanwhile, we understand that the pyrolysis method provides a smaller size of carbon dots with a higher quantum yield than other synthetic methods. The fluorescence property of carbon dots is highly dependent on the size and surface oxidation or surface passivation. These salient findings provide a vast opportunity to develop CDs-based scaffolds in basic research and biomedical applications. Though many potential research works are carried out regarding the application of CDs in tissue engineering, there are still few challenges needed to address under this topic, for example:

(1) Preparation of highly performing CDs with suitable size, morphology, crystallinity, and number of surface functional groups should be optimized through a systematic and scalable synthetic protocol. Besides, creating in situ technique is essential to understand the mechanism of CDs’ formation.
(2) Many experiments and theoretical modeling are essential to thoroughly understand the relationship between the optical properties with methods of synthesis of carbon dots.
(3) Biomedical researchers have paid attention to the excitation of CDs from red to NIR region (i.e., 600–1000 nm), which contributes to significant light scattering and deeper light penetration into biological tissues, reducing loss of photon energy, and increase in CDs’ biomedical efficacy. However, the preparation of red emissive CDs is a challenging task with very few synthetic methods and the associated indirect bandgap narrowing mechanisms. To mitigate this problem, future research works should be carried out focusing on developing red-to-NIR excitable carbon dots.
(4) Fabrication of next-generation carbon dot-based scaffolds compatible with the surrounding tissues after implantation is essential to minimize clinical trial errors.
(5) When the fluorescent CDs are buried into the scaffold material, the fibers’ fluorescence emission intensity is generally weakened. Further, some scaffold-CDs may suffer leaching issues depending on the nature of the polymer used for the scaffold. Such a problem would result in a decrease in the fluorescent probe concentration in the scaffold and decrease the emissive photon signals. Hence, a detailed study should be presented to amplify the application of CDs in tissue regeneration considering the above problem.
(6) Different synthetic approaches imparting hydrophilicity and surface functionalization are reasonable for the lower cytotoxicity of the synthesized carbon dots. Hence, the researchers should evaluate the cytotoxicity of carbon dots alone before blending them with suitable scaffolds.
(7) Different non-invasive imaging methods having their advantages and disadvantages in clinical trials limit their application in the tissue engineering field. Hence, developing carbon dots mediated tissue engineering scaffold with multimodal imaging capability will enhance their potential in clinical studies.

We expect that the continued research works of fluorescent carbon dots mediated scaffolds for the next several years will advance the field of non-invasive tissue engineering by dealing with the existing limitations.

## Figures and Tables

**Table 1:** A list of synthetic approaches of carbon dots from various precursors and their size, quantum yield, and applications.

Synthetic Methods		Carbon Source	Reaction Condition	Size of CDs (nm)	Quantum Yield (%)	Application	Study Authors
Top-down approaches	Arc discharge deposition	Arc soot	Electrodes, agarose gel	1	1.6	-	Xu et al. (2004) [64]
	Arc discharge deposition	oxCNT	Electrodes	-	-	-	Bottini et al. (2006) [65]
	Arc discharge deposition	Graphite	Electrodes, current 100–150 A	4–6	2.3–8.7	-	Dey et al. (2014) [66]
	Laser ablation	Graphite powder and cement	Nd:YAG laser (1064 nm, 10 Hz)	5	4–10	-	Sun et al. (2006) [72]
	Laser ablation	Graphite powder	Nd:YAG laser (wavelength 1.64 µm, intensity 6.0 × 10^6^ W cm^−2^), 2 h	3.2–3.3	3–8	-	Hu et al. (2009) [73]
	Laser ablation	Nano-carbon material	Nd:YAG laser wavelength 532 nm	-	-	-	Li et al. (2011) [67]
	Electrochemical carbonization	Alcohols	Current density 15–100 mA cm^−2^, 4 h	2.1–4.3	15.9	Bioimaging	Deng et al. (2014) [74]
	Electrochemical carbonization	Amino acids	1–10 V, 2 h	2.95 ± 0.12	46.2	Cellular imaging, Fe^3+^ ions detection, and fiber staining	Niu et al. (2017) [69]
	Electrochemical carbonization	Carbon fibers	0.5–2.5 V, 2 h	2.2 ± 0.6–3.3 ± 0.6	1.47 ± 0.20	-	Bao et al. (2011) [75]
	Ultrasonication	Food waste	20 kHz, 45 min	4	2.85	Bioimaging	Part et al. (2014) [77]
	Ultrasonication	Activated carbon	40 kHz, 2 h	5–10	5	-	Li et al. (2011) [78]
	Ultrasonication	PEG-400	20 kHz, 230 V AC, 3 h	6	-	Catalyst	Maruthapandi et al. (2018) [70]
Bottom-up approaches	Hydrothermal	Urea, p-phenylenediamine	160 °C, 10 h	2.6	35	Bioimaging	Ding et al. (2016) [80]
	Hydrothermal	Orange juice	120 °C, 2.5 h	2.5	26	Cellular imaging	Sahu et al. (2012) [81]
	Hydrothermal	Chitosan	180 °C, 10 h	2–10	6.6	Metal ion detection	Zhan et al. (2019) [82]
	Hydrothermal	Citric acid, ethylenediamine	150–300 °C, 5 h	2–6	80	Printing ink, biosensor, and composites	Zhu et al. (2013) [83]
	Microwave	PEG-200 and saccharides	500 W, 2–10 min	2.75 ± 0.45–3.65 ± 0.6	3.1–6.3	-	Zhu et al. (2009) [90]
	Microwave	Glycerol and TTDDA	700 W	3.5	12.02	Cellular imaging	Liu et al. (2011) [91]
	Microwave	Lemon and onion biomass	1450 W, 6 min	6.15	23.6	Detection of riboflavin	Monte-Filho et al. (2019) [92]
	Microwave	Gelatin and PEG	600 W, 10 min	6	34	Bioimaging and anticancer	Arsalani et al. (2019) [84]
	Microwave	Aspirin and hydrazine	500 W, 8 min	2–5.5	23	Cellular imaging and bioimaging	Xu et al. (2016) [93]
	Solvothermal	Spinach	150 °C, 6 h	3–11	15.34	Bioimaging	Li et al. (2017) [95]
	Solvothermal	1,3,6-trinitropyrene	230 °C, 12 h	2.25–3.98	6.4–59	Bioimaging	Zhan et al. (2018) [85]
	Reverse micelles	CCl_4_, TOAB, and allylamine	Stir for 30 min	2–6	27	-	Linehan et al. (2014) [86]
	Solvothermal assisted reverse micelles	DS and Brij L4	Stir for 30 min, 180 °C, 10 h	5 ± 0.7	2.6	-	Prikhozhdenko et al. (2018) [97]
	Reverse micelles	Glucose and AOT	160 °C	~2–4	35	-	Kwon et al. (2012) [98]
	Pyrolysis	Glycerol and PEG	230 °C, 30 min	5.5 ± 1.1	20	Cellular imaging and drug delivery	Lai et al. (2012) [99]
	Pyrolysis	DTPA	180 °C	5	17	Detection of curcumin	Shi et al. (2015) [87]
	Pyrolysis	CA, EDA	150 °C, 20 min	5	88	-	Yin et al. (2019) [101]

**Table 2:** Fabrication of various carbon dots-based tissue engineering constructs, physicochemical properties, and their biomedical application and salient features are shown.

Composite of CQDs	Tissue Regenerative Scaffold and Methodology	Physicochemical Properties	Invasive/Non-Invasive Imaging Devices and Performances	Cellular Type	Salient Observations	Biomedical Applications	Study Authors
SF-CQD-PLA	Electrospun nanofiber	Higher porosity, better swelling ability, and Young’s modulus of 1610 kPa	CLSM/Cell proliferation	H9c2 rat myoblast cells	Increased cell viability and cardiac-maker gene expression	Tissue engineering and nursing care	Yan et al. (2019) [49]
CP-CQD-PCL	Electrospun nanofiber	Decreased fiber diameter, increased surface hydrophilicity, and Young’s modulus of 2.83 ± 0.23 MPa	SEM/Cell morphology and adhesion	MG-63 human osteoblast cells	Increased cell proliferation and ALP activity; more drug release at lower concentration	Bone tissue engineering	Ghorghi et al. (2020) [108]
CMCS-CQD_AG_-OD_ex_	Injectable hydrogel/Chemical cross-linking method	Self-healing, stretchable, compressive property, pH dependent release of CQDs, and compressive modulus of 11.441 kPa	Fluorescence and optical microscopy/Cell adhesion survival and RBC imaging	NIH-3T3 mouse embryonic fibroblast cells	Inhibition of biofilm formation; low drug resistance; better antibacterial and anti-inflammation; higher collagen deposition	Skin tissue regeneration	Li et al. (2021) [112]
BC-CQDs-Si NPs-SF& PVA-CQDs-Si NPs-SF	Nanofiber/Spray printing and electrospinning method	Moderate swelling, biodegradability, and Young’s modulus of 320 MPa & 70 MPa	Optical microscopy/Cell migration and tissue regeneration	NIH-3T3 fibroblasts	Better antibacterial activity; enhanced cell viability, proliferation, and re-epithelialization	Skin tissue and hair regeneration	Abolghasemzade et al. (2021) [110]
PCL-CNDs-gelatin	Electrospun nanofiber	Fiber diameter of 698 ± 420 nm, pore diameter of 2.93 ± 1.13 µm, photo stability, and Young’s modulus of 0.866 ± 0.06 MPa	SEM and two-photon microscopy/Cell attachment, growth, cytotoxicity, and skin regeneration kinetics	Human fibroblasts and keratinocyte cells	Increased antioxidant activity; accelerated cell adhesion, migration, and proliferation	Skin tissue regeneration	Pal et al. (2017) [146]
CQDs-chitosan	Film/Solvent casting method	Decrease in water absorption and moisture permeation and 1.3-fold flexibility	-	-	Enhanced antioxidant activity	Skin tissue regeneration and drug delivery	Kandra et al. (2020) [147]
PCL-PVA-TCP-CD	Electrospun nanofiber	Fiber diameter range of 300–414.8 nm and contact angle of 33–45°	SEM and CLSM/Cell attachment, morphology viability, and proliferation	Human buccal fat pad-derived stem cells (hBFPSCs)	Enhanced ALP activity, osteogenic differentiation, and cell proliferation	Bone tissue engineering	Shafiei et al. (2019) [145]
CDP-*f*-PU	Injectable hydrogel/Chemical cross-linking method	Moderate biodegradation	Phase contrast and CLSM/Cell viability, adhesion, migration, morphology, and vascularization pattern	MG-63 human osteoblasts	Better biocompatibility, osteoblast adhesion, proliferation, and differentiation	Bone tissue engineering	Gogoi et al. (2017) [148]
CD-HAp-PU	Film/Chemical cross-linking method	Dose dependent thermal stability and Young’s modulus of 28.42 ± 0.35 MPa	Fluorescence microscopy/Cell morphology, viability, and proliferation	MG-63 human osteoblasts	Higher cytocompatibility, cell proliferation, and ALP activity	Bone tissue engineering	Gogoi et al. (2016) [116]
Chitosan-CDs	Hydrogel/Solvent casting method	Higher pH sensitivity and Young’s modulus of 2.90 ± 0.15 GPa	Fluorescence microscopy/Cell viability and proliferation	L929 mouse fibroblasts	More antibacterial activity; better biocompatibility	Skin tissue regeneration	Omidi et al. (2017) [109]
PLA-CQDs	Scaffold/3D Printing method	Long term photostability and pH flexible fluorescence	Two-photon & multi-photon microscopy/Cell-scaffold imaging	RL-14 human fetal ventricular cardiomyocyte cell	Excellent biocompatibility; deep cellular and scaffold imaging	Tissue engineering	Dehghani et al. (2018) [26]
SA-Hb-CQDs	Injectable hydrogel/Phase separation	Contact angle of 42.5°, higher pH sensitivity, and excellent biodegradability	CLSM/Cell viability	NIH-3T3 fibroblasts	Enhanced biocompatibility and antibacterial activity	Skin tissue regeneration	Zhang et al. (2020) [149]

## Data Availability

No new data were created or analyzed in this study. Data sharing is not applicable to this article.

## Abbreviations

CLSM	Confocal laser scanning microscopy
NIH-3T3	Mouse embryonic fibroblasts cells
DS	Dextran sulfate sodium salt
BrijL4	Polyoxyethylene (4) lauryl ether
AOT	Bis(2-ethylhexyl)sulfosuccinate sodium salt
DTPA	Diethylenetriaminepentaacetic acid
CA	Citric acid mono hydrate
EDA	Ethylenediamine
OxCNT	Oxidized carbon nanotube
PCL	Polycaprolactone
CP	Captopril
BC	Bacterial cellulose
PVA	Poly(vinyl alcohol)
SF	Silk fibroin
PU	Polyurethane
PLA	Poly(L-lactic acid)
PP	Polypropylene
H9C2	Cardio myoblast cell
MG-63	Human osteoblast cell
3T3	Mouse fibroblasts cell
hBFPSCs	Human pluripotent stem cell registry
ALg	Alginate
CNF	Cellulose nanofibers
HAp	Hydroxyapatite
ALP	Alkaline phosphatase
CMCS	Carboxymethyl chitosan
FL	Fluorescence
CQD_AG_	Antibacterial carbon quantum dots
RBC	Red blood cell
TTDDA	4,7,10-trioxa-1,13-tridecanediamine
